# Traumatic Spigelian Hernia Following Blunt Abdominal Trauma

**DOI:** 10.7759/cureus.35564

**Published:** 2023-02-27

**Authors:** Boris Kangabam

**Affiliations:** 1 Urology, Regional Institute of Medical Sciences, Imphal, IND

**Keywords:** handlebar injury, pediatric, abdominal wall hernia, spigelian hernia, trauma

## Abstract

Traumatic abdominal wall hernia (TAWH) is a rare form of hernia occurring after blunt trauma to the abdomen. Traumatic Spigelian hernia is an uncommon subtype sporadically described in the literature. It is an anterior abdominal wall defect occurring along the Spigelian aponeurosis bounded laterally by the semilunar line and medially by the rectus abdominis muscle. Imaging with CT is the investigation of choice. The surgeon has a variety of treatment options ranging from the traditional midline laparotomy to laparoscopic repair with or without the use of mesh. Conservative treatment has also been advocated as a safe and feasible option in select cases. Described here is a case of traumatic Spigelian hernia following blunt abdominal trauma caused by a motorcycle handlebar in a 17-year-old male.

## Introduction

Traumatic abdominal wall hernia (TAWH) is an uncommon hernia occurring in less than 1% of blunt abdominal trauma first described by Selby in 1906 [1]. It is an abdominal wall defect produced by a blunt force that disrupts the muscle and fascia with the overlying skin remaining intact. Traumatic Spigelian hernia is an uncommon subtype and it has been reported sporadically in the literature. Spigelian hernia is a defect in the anterior abdominal wall occurring along the Spigelian aponeurosis bound medially by the rectus abdominis muscle and the semilunar line laterally. It may be congenital or acquired. The first description of traumatic Spigelian hernia came in 1933 despite the high incidence of blunt abdominal trauma [2]. The diagnosis of Spigelian hernia is clinical though it can be challenging due to minimal physical findings. A radiological investigation is a useful adjunct in making the correct diagnosis. Surgical repair remains the mainstay of treatment. The decision for immediate or delayed repair with or without the use of mesh is individualized to each patient depending on the presentation, history, and presence of concomitant intra-abdominal injuries. Choice of mesh, whether biological or synthetic, may be chosen depending on the level of contamination of the wound. This report features a case of isolated traumatic Spigelian hernia in an adolescent male following a motorcycle handlebar injury in a road traffic accident that was diagnosed based on its location in the Spigelian aponeurosis.

## Case presentation

**Figure 1:**
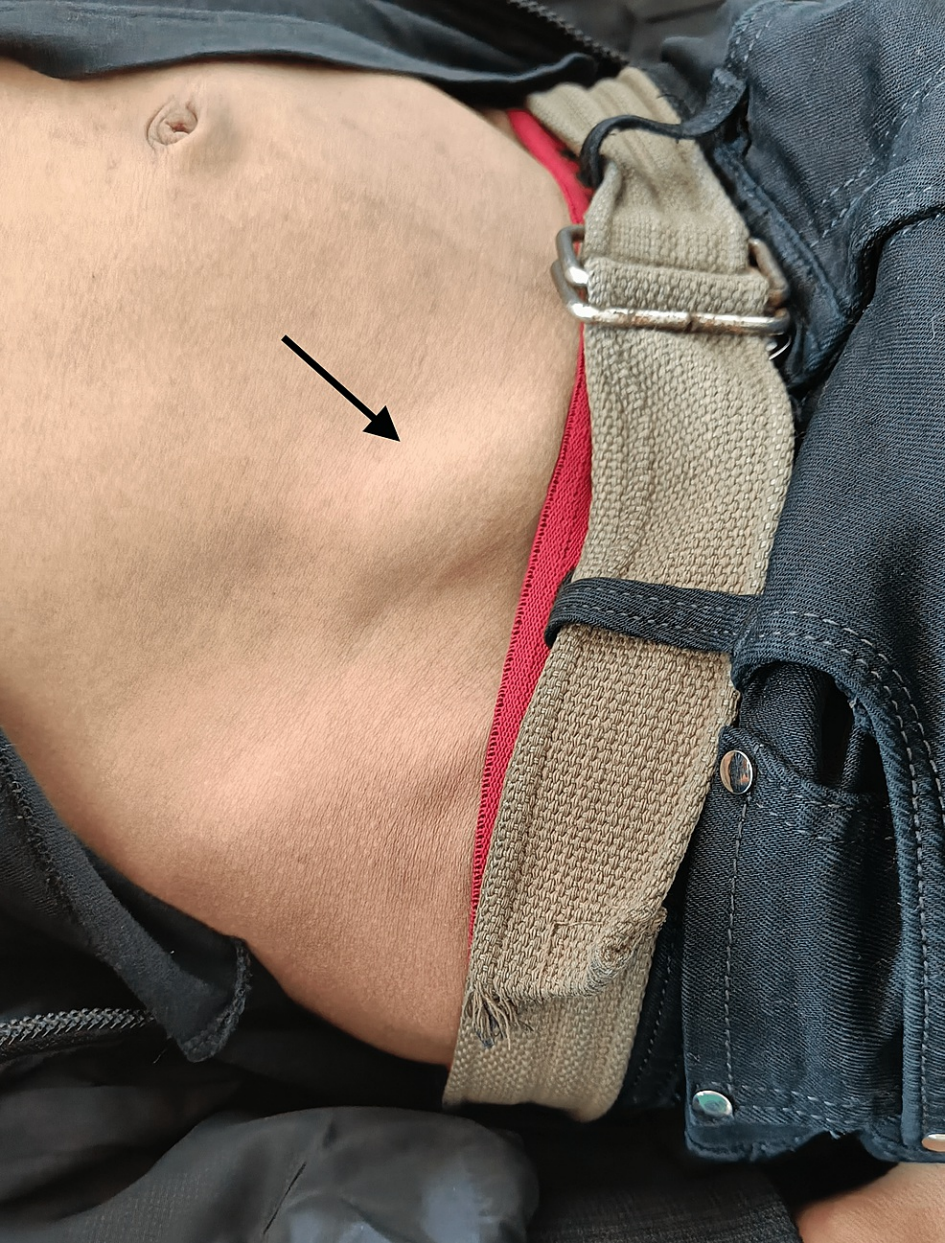
Right lower abdomen swelling made more prominent with the Valsalva maneuver indicated by the black arrow

A 17-year-old male presented to the emergency department two hours after abdominal trauma caused by the tip of the motorcycle handlebar end at the right lower abdomen with complaints of a reducible swelling in the right lower abdomen (Figure 1). On investigating the details of the accident, it was found that the patient was riding a motorcycle when he lost control, hit his abdomen on the end of the motorcycle handlebar, and subsequently fell to the ground. He immediately noticed the swelling, which was initially painful for approximately 20 minutes but the pain subsided gradually on its own without any medications. Upon arrival at the emergency room, the patient was fully conscious and could walk on his own without difficulty. The patient was not wearing any protective equipment such as a helmet while he was riding. He had no history of nausea, vomiting, loss of consciousness, seizure, blurring of vision, or bleeding from any site. He had no history of abdominal pain or swelling prior to the accident. He was a non-smoker and had no significant medical or surgical history in the past. On examination, the patient was conscious, cooperative, and well-oriented to time, place, and person. His vitals were hemodynamically stable with a blood pressure of 114/70 mm Hg measured in the left arm in a sitting position, a pulse of 76 beats per minute, and a respiratory rate of 16 breaths per minute. There was a reducible, non-tender swelling measuring around 5 cm × 4 cm in the right lower abdomen. There was no local rise in temperature and no overlying skin changes like abrasion or ecchymosis. A cough impulse was present and a defect of around 1 cm could be felt in the anterior abdominal wall underneath the swelling. The swelling increased on standing and coughing and decreased on lying down. There was no abdominal distention or rigidity. Routine blood and urine investigations were done and found to be within normal limits. The extended focussed assessment with sonography in trauma (eFAST) scan was negative.

**Figure 2:**
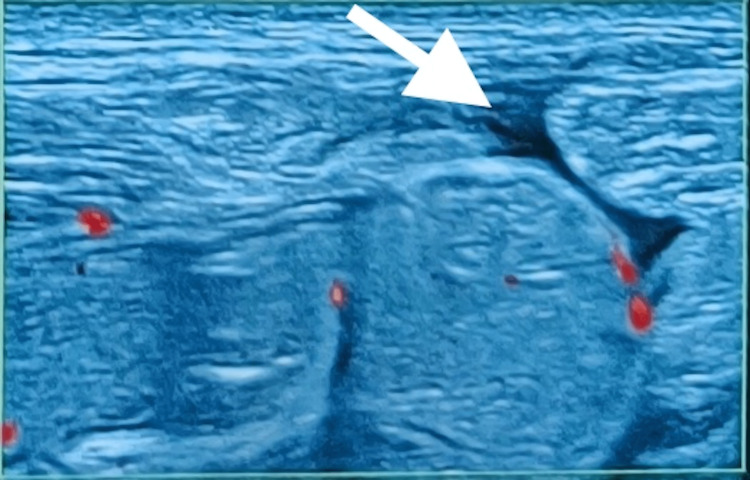
Ultrasonography of the right lower abdominal wall shows the abdominal wall defect on the longitudinal plane indicated by the white arrow

**Figure 3:**
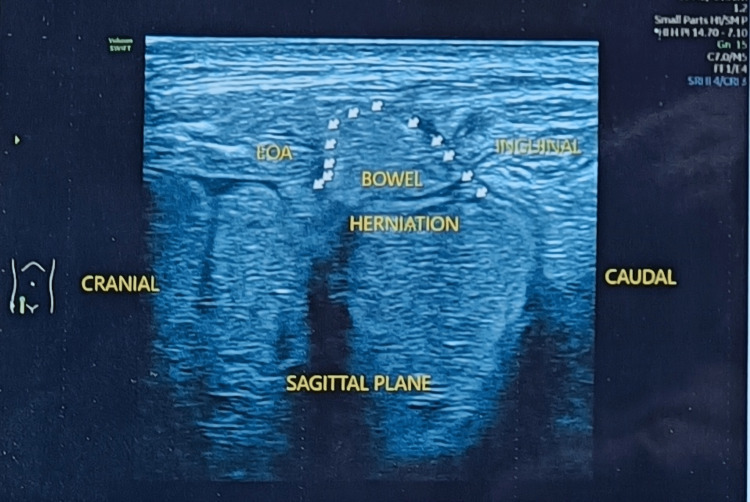
Ultrasonography of Spigelian hernia performed while in a standing position shows herniating bowel loops (EOA) indicated by multiple small white arrows EOA: External oblique aponeurosis

**Figure 4:**
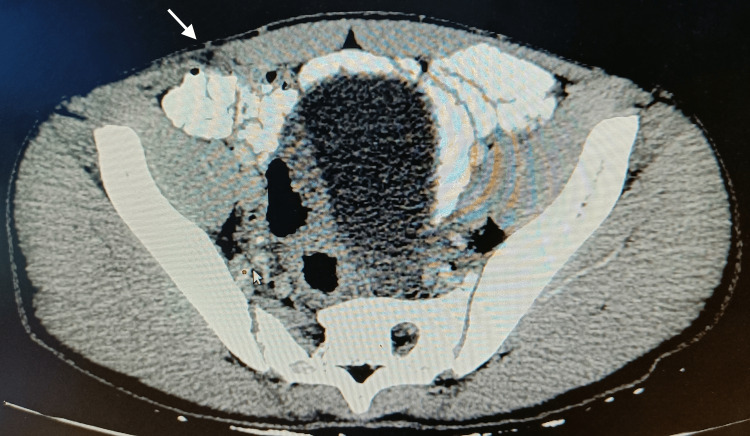
Contrast-enhanced CT abdomen cross-sectional view shows a defect in the Spigelian fascia lateral to the right rectus abdominis muscle indicated by a white arrow

Abdomen ultrasonography revealed a defect of 1 cm × 1 cm in the right lower abdomen along the right Spigelian aponeurosis lateral to the rectus abdominis muscle and medial to the semilunar line (Figure 2). Herniating bowel loops were visualized on standing (Figure 3). Contrast-enhanced CT of the abdomen showed a 1 cm defect lateral to the rectus muscle and no other intraabdominal injury (Figure 4). The patient was diagnosed to have a reducible, uncomplicated right-sided Spigelian hernia following blunt abdominal trauma with bowel loop as hernial contents. He was offered surgery but the patient declined. The patient did not have any complaint related to the swelling over the last six months and was advised regular follow-up on an outpatient basis.

## Discussion

Damschen et al. defined TAWH as a hernia appearing immediately after a blunt trauma through the muscle and fascial layers with the overlying skin intact [3]. Traumatic Spigelian hernia is an uncommon subtype of TAWH. The Spigelian hernia was first described by the Belgian anatomist Adriaan Van Der Spiegel, as a hernia occurring through the Spigelian fascia [4]. Spigelian hernias make up about 0.1% to 2% of all abdominal wall hernias. However, there are few case reports of traumatic Spigelian hernia [4]. Notably, the majority of traumatic Spigelian hernia cases reported were in males. The Spigelian hernia has a tendency to develop in chronically weakened abdominal walls and factors contributing to high intraabdominal pressure. Some common factors include multiparity, chronic cough, obesity, and comorbidities like diabetes mellitus, peripheral vascular disease, chronic obstructive pulmonary disease, stroke, and myocardial infarction [4].In contrast, traumatic Spigelian hernia appears acutely following trauma like handlebar injuries, falls from height, etc. [5]. Wood et al. in 1988 attempted to classify TAWH into three types based on the mechanism of injury and size of the defect. Abdominal wall defects sustained from high-energy injuries like a motor vehicle accident or a fall from height were classified as type I, and defects following low-energy injuries such as bicycle handlebar injury as type II. Type III, being the least common, features defects with intra-abdominal herniation of bowel loops following deceleration injuries [6]. Our case according to this classification can be grouped under type I TAWH based on the high-energy injury caused by the motorcycle handlebar and the relatively smaller size defect of 1 cm.

The pathophysiology of TAWH occurs with a sudden rise in pressure inside the abdominal cavity following blunt trauma to the abdomen. The force spreads diffusely over the abdomen without resulting in penetration of the abdominal skin. Following this increase in intraabdominal pressure, hernia tends to develop in anatomically weak points [6]. A Spigelian hernia occurs lateral to the semilunar line through the Spigelian aponeurosis. It tends to occur in the Spigelian belt, an imaginary 6 cm wide band above the inter-spinous line [7]. Notably, the posterior rectus sheath is absent in the lower half of the abdomen below the arcuate line [5]. Clinical features include the sudden appearance of a palpable mass following trauma. The overlying skin is intact although it may be associated with abrasion or ecchymosis [5]. It may be associated with bowel obstruction or strangulation. Hernia, if not apparently visible, may be made more prominent with the Valsalva maneuver and while in a standing position. However, this may be difficult to do in trauma patients [5].

Spigelian hernia is diagnosed clinically. However, imaging may be done as a useful adjunct. Ultrasound is a cost-effective, readily available, conclusive imaging tool with a sensitivity of 83% to 90% [8]. It provides dynamic evaluation with the ability to see the underlying hernial contents in a short period of time. However, it is operator dependent. Contrast-enhanced CT is the investigation of choice that allows the evaluation of concomitant visceral injuries. Diagnostic laparoscopy is also an armamentarium for the surgeon. It allows for local repair of hernia when there are no other intra-abdominal injuries, avoiding laparotomy.

Many studies advocate immediate midline laparotomy due to concomitant intra-abdominal injury in 30% of cases. Mesh repair of Spigelian hernia is durable and has a low recurrence rate of 4.3% . The choice of mesh may be synthetic or biological depending on the contamination of the wound. Mesh-free laparoscopic repair with transfascial suture techniques is also another option when the hernia is small. Conservative treatment may be considered in patients with multiple comorbidities, asymptomatic hernias, large hernias with a low chance of strangulation, and no associated intra-abdominal injuries [9].

## Conclusions

Traumatic abdominal wall hernia should be suspected in cases with a sudden appearance of swelling following blunt abdominal trauma. There may be isolated traumatic Spigelian hernia without concomitant intraabdominal visceral injury. The TAWH tends to develop in anatomically weak points with a sudden rise in intra-abdominal pressure following blunt trauma to the abdomen irrespective of the site of impact. Ultrasound provides dynamic imaging in reducible swellings employing position change and the Valsalva maneuver. A CT with contrast is the investigation of choice in confirming TAWH. Treatment is tailored according to the patient's mode of injury, presentation, and associated comorbidities. Conservative treatment with a plan for delayed operative repair may be tried in isolated TAWHs, though surgery is preferred in cases with concomitant visceral injuries.
